# Effect of Host Cholesterol on the Membrane Dynamics of Outer Membrane Lipids of Mycobacteria

**DOI:** 10.1002/asia.202300697

**Published:** 2023-10-27

**Authors:** Pranav Adhyapak, Kuan Liang, Mojie Duan, Shobhna Kapoor

**Affiliations:** [a] Department of Chemistry, Indian Institute of Technology Bombay, Mumbai 400076 (India); [b] National Centre for Magnetic Resonance in Wuhan, State Key Laboratory of Magnetic Resonance and Atomic and Molecular Physics, Innovation Academy for Precision Measurement Science and Technology, Chinese Academy of Sciences, Wuhan 430071 Hubei (China)

**Keywords:** Mycobacterial membrane lipids, Membrane order, Phase-segregated lipid domains, Cholesterol, Latent Tubercular infection, Membrane-drug interactions

## Abstract

The ability of *Mycobacterium tuberculosis* to remain dormant after primary infection represents the prime cause of new TB cases throughout the world. Hence, diagnosis and treatment of individuals hosting dormant mycobacterium is one of the crucial strategies to be adopted for the prevention of Tuberculosis. Among many strategies unleashed by the latent bacterium, one of them is scavenging host cholesterol for carbon source. Cholesterol modifies lipid membranes over many scales and here, its effect on mycobacterial membrane biophysics and the subsequent effect on partitioning of antibiotics into cholesterol- enriched mycobacterial membranes was investigated. Our research showed that cholesterol alters the phase state behavior of mycobacterial outer membrane lipids by enhancing the overall membrane order at the head-group and acyl chain region and is integrated into both ordered and disordered domains/phases, with a preference for the latter. Exogenous cholesterol further alters the drug partitioning behavior of structurally different drugs, pointing to a larger clinical potential of using more hydrophobic medications to target dormant bacteria.

## Introduction

The amazing ability of *Mycobacterium tuberculosis* (*Mtb*) to adapt and endure the host is a contributing factor in its success as a pathogen.^[1,2]^ Activated macrophages prevent intracellular *Mtb* from proliferating by denying them vital nutrients and confining them with cellular clusters called granulomas.^[3]^ Inside granulomas, the bacterium needs to find means to obtain nutrients for intracellular survival and growth. Furthermore, the challenges faced by the bacterium inside the host keeps changing with disease progression.^[4]^ For instance, in the latent stage of infection, *Mtb* infected macrophages are sequestered in granuloma under hypoxic condition containing TAG (Triacyl glycerol), cholesterol and other fatty acid rich lipid droplets, which provide lipid rich environment for *Mtb*.^[5–7]^ Furthermore, during infection in humans and mouse models, *Mtb* has been found in lipid-rich lesions and its genome contains large number of genes involved in β-oxidation. This highlights the importance of lipid catabolism.^[8,9]^

**Figure 1:**
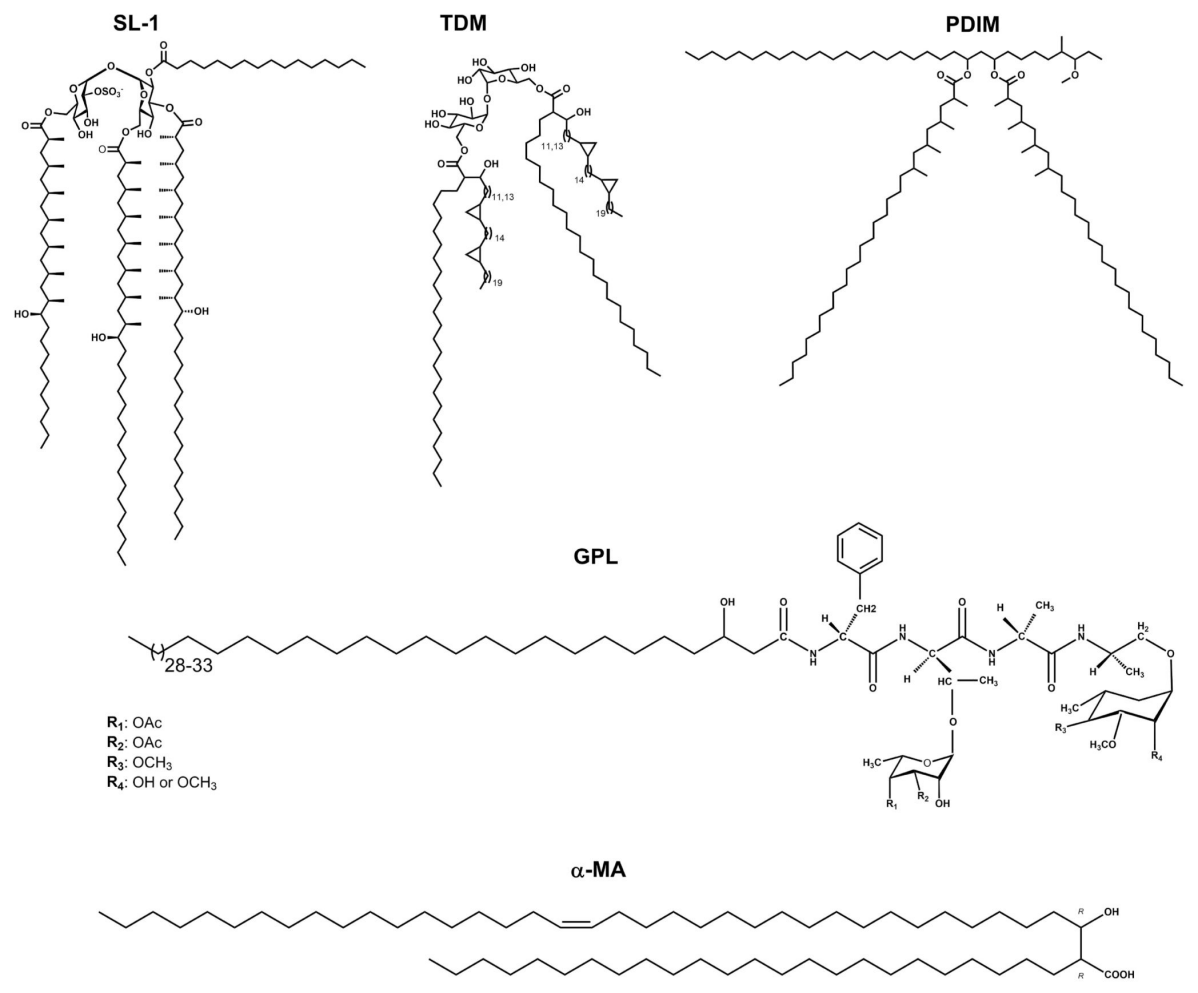
Structures of representative lipids present in the outer membrane of *Mycobacterium tuberculosis*.

Under such conditions, *Mtb* changes its carbon diet to cholesterol (Chol) to derive both carbon and energy from this essential component of host membranes.^[6,7,10]^ Furthermore, during specific stages of infection, the ingested bacteria exploits both active and passive cholesterol import pathways, including cholesterol diffusion through the host cell plasma membrane.^[11,12]^ *Mtb* encodes *mce* operons that help in recruiting cholesterol into the bacterium via transmembrane cholesterol transporters.^[13,14]^ However, in the absence of some of these transporters, substantial cholesterol accumulation inside bacterial cells strongly implicates entry of host cholesterol by diffusion through the bacterial cell membrane.^[10,11]^ In this regard, interactions of cholesterol with the mycobacterial cell membrane have not been studied and further compounded by a complex bacterial membrane architecture with most intriguing lipids localized in the outer membrane layer of the cell envelope. They consist of structurally and chemically diverse lipids such as long chained (C_60_–C_90_) mycolic acids (MA), glycolipids such as GPLs (glycopeptidolipids), TDM (trehalose dimycolate) having MA chains and cyclopropane rings, PDIM (phthoicerol dimycocerosate) and (sulfolipids) SLs having methyl branches and hydroxylated kinks.^[15,16]^ (Figure 1).

The unexplored interaction landscape of cholesterol with mycobacterial outer lipids is expected to impact the bacterial membrane composition and properties eventually affecting cholesterol passage, bacterial physiology and drug interactions. Thus, exploring how cholesterol affects the shape, phase state, and function of the outer membrane lipids (OML) of *Mtb* cell envelope is warranted. This is further emphasized by the duration of the latent stage infection (2 to 5 years^[17]^) wherein, the bacteria recruits host cholesterol from host cell. This exogenous cholesterol in the *Mtb* membrane may aid the bacterium in evading drug action; inability of INH (isoniazid) and moxifloxacin to target persistent bacteria.^[18,19]^ This prompted us to investigate how cholesterol affects the membrane dynamics of the Mycobacterium cell envelope using time resolved fluorescence spectroscopy, FLIM (Fluorescence Lifetime Imaging Microscopy), AFM (Atomic Force Microscopy). Additionally, we examined the drug partitioning of rifampicin (RIF) and moxifloxacin (MOX) into OML in the presence of exogenous cholesterol in order to better understand the role of cholesterol in controlling drug-membrane interaction. Collectively, the research showed that cholesterol modulates the phase state behaviour of OML. Exogenous cholesterol increased membrane order at the headgroup and acyl chain area via partitioning into distinct membrane domains of OML. Furthermore, exogenous cholesterol inhibited the partitioning of MOX but enhanced that of RIF. This work provides a molecular picture of the host-cholesterol and bacterial membrane interactions and its impact on drug partitioning revealing therapeutic insights on targeting persistent bacteria.

## Results and Discussion

### Impact of exogenous Cholesterol on mycobacterial membrane interfacial hydration and fluidity

**Figure 2:**
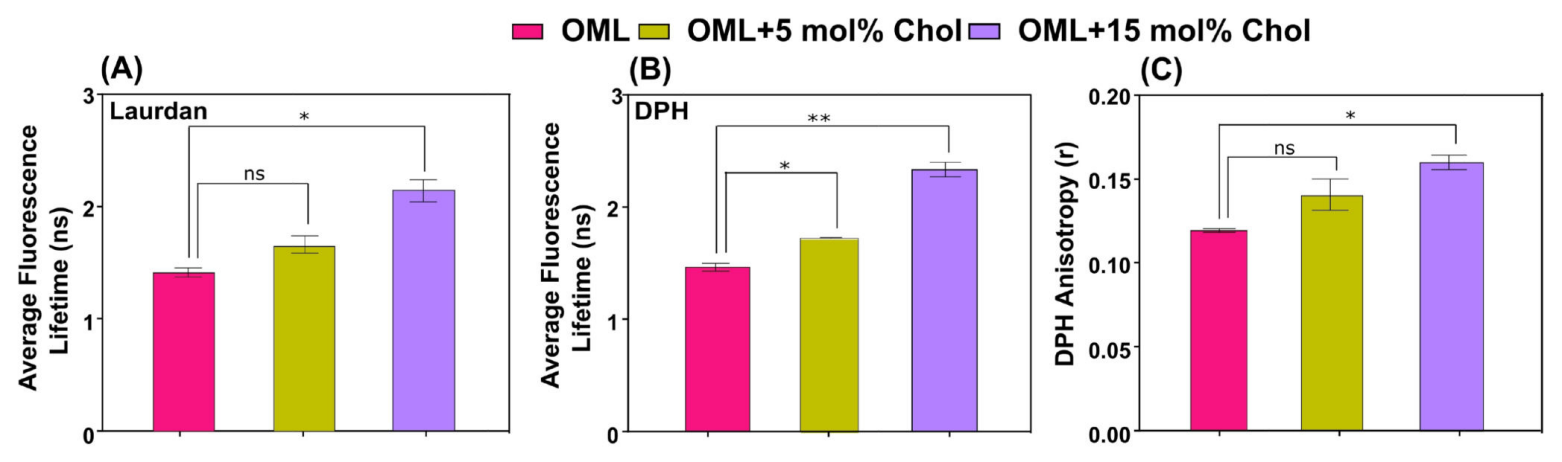
Cholesterol-dependent changes in order and fluidity of OML measured using steady-state and time-resolved fluorescence spectroscopy. (A–B) Average fluorescence lifetime of Laurdan and DPH (360 nm excitation) measured at 430 nm emission wavelength in OML with or without addition of 5 and 15 mol% cholesterol. (C) Steady-state fluorescence anisotropy of DPH in indicated lipid systems. DPH was excited at 352 nm and anisotropy calculation was performed using 430 nm (emission maxima). All the measurements were carried out at 23 ± 1 °C. Data represented as mean ± SD of 3 independent experiments. Significance was determined using one-way ANOVA with Tukey’s multiple comparison test.

To investigate interactions of cholesterol (Chol) and mycobacterial lipid membrane, protein-free fractions of OML from *Mycobacterium smegmatis* (a lab model for *Mtb*), *Msm* was extracted by our previously reported procedure.^[15,20]^ Thin layer chromatography (TLC) revealed selective extraction and identification of characteristic outer membrane lipids, (OML) (Figure S1). Quantitative estimation of outer membrane lipids was studied using LC–MS/MS (Fig S2). Ac_1_PIM_1_ (monoacylated phosphoinositol mannosides), glycopeptidolipids and glycerol-based lipids (monoacyl glycerol, diacylglycerol and phosphatidyl glycerol) were found to be most abundant lipid in OML. Whereas, diacyl trehalose, cardiolipin and other lysophospholipids were present in low abundance. Next, three different *in vitro* membrane models were designed: OML only, OML + 5 mol% Chol, OML + 15 mol% Chol. The rationale for choosing the indicated cholesterol mol% stems from the previous study.^[6]^ Time-resolved fluorescence spectroscopy using TCSPC (time correlated single photon counting) with Laurdan allowed probing the interfacial hydration pattern of OML in absence and presence of exogenous Chol. An increase in the fluorescence lifetime of Laurdan indicates that Chol increases the headgroup packing of OML (Figure 2A and Table 1). Next, DPH lifetime reported a similar trend for the acyl chain dynamics of OML (Figure 2B), underlining an increase in acyl chain ordering. Next rotational mobility of DPH using steady-state anisotropy showed an increase in anisotropy (*r*) upon Chol addition indicating a hindered rotational mobility of the probe and hence decreased fluidity of OML (Figure 2C). Overall, these measurements show that Chol insertion into OML increases the membrane ordering both the headgroup and acyl chain region in OML.

### Effect of Cholesterol on lateral phase organization and nanomechanics in OML

**Figure 3:**
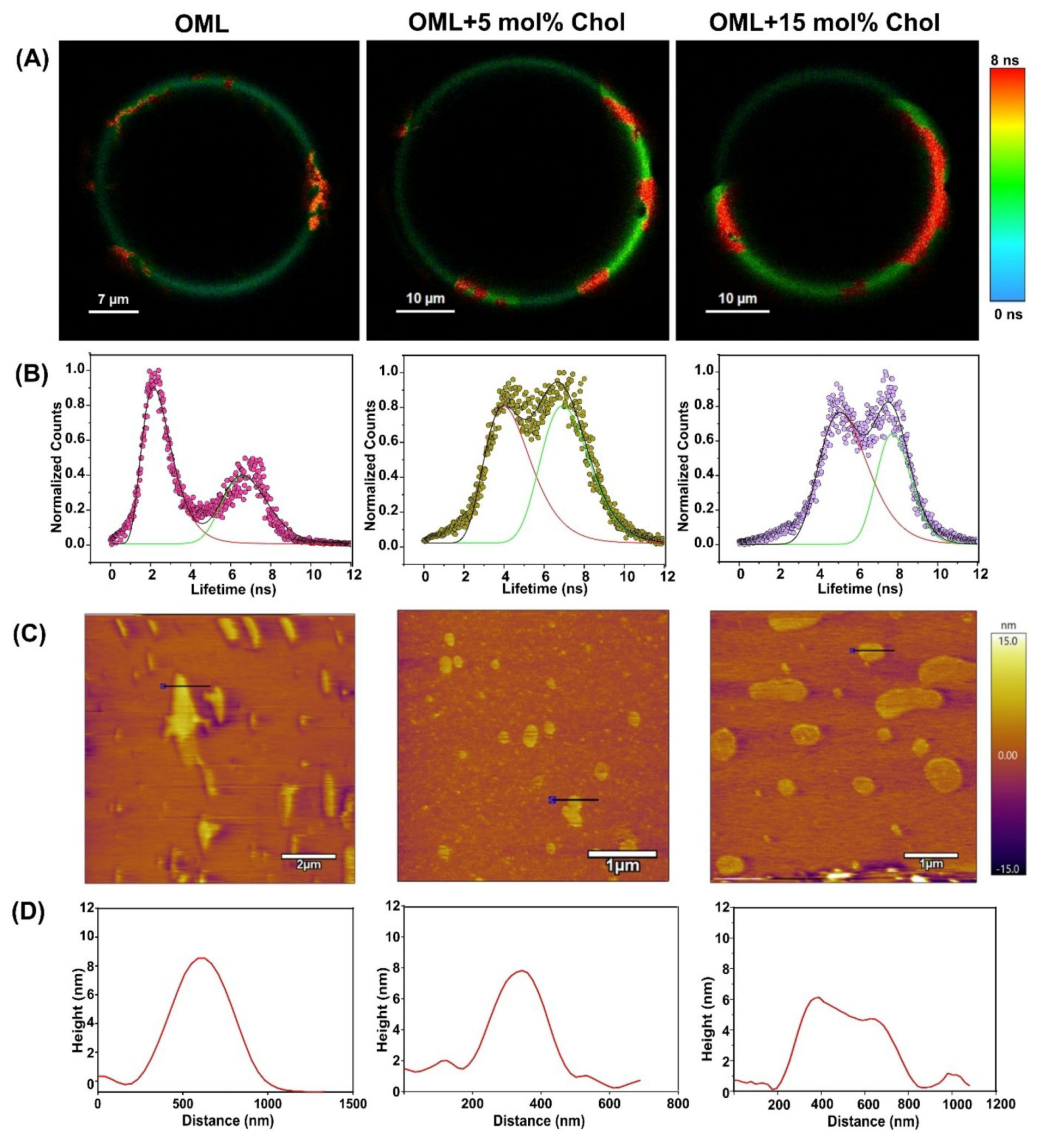
Visualization of phase state behaviour of OML with addition of 5 and 15 mol% cholesterol by using FLIM (A) and its lifetime distribution histogram (B). For FLIM, Laurdan (405 nm excitation) was used as a fluorescent probe. Laurdan emission was collected from 430–490 nm. The lifetime distribution was fitted with lognormal function using Origin Pro 2018b. AFM topography 2D images of indicated membrane systems (C) and the height profile analysis (D) of the region highlighted as black line in AFM images. All the measurements were carried out at 23 ± 1 °C.

To visualize the phase state behavior of OML as a function of Chol, we prepared Laurdan labelled GUVs (Giant Unilamellar Vesicles). Fluorescence lifetime imaging microscopy (FLIM) rendered visualization of membrane domains (Figure 3A), and demonstrated lifetime segregated lipid domains in all lipid systems. The green colored domains of low lifetime likely represent the liquid-disordered (*L*_*d*_) domains and the orange-red colored domains of higher lifetime likely represent the liquid-ordered (*L*_*o*_) phase in OML GUVs. Fluorescence lifetime distributions (Figure 3B) was deconvoluted using lognormal fitting function into two components denoting the two-phase segregated domains, i. e., *L*_*d*_ (low lifetime) and *L*_*o*_/Gel (high lifetime). Presence of exogenous Chol increased the population of high Laurdan lifetime pixels, i. e., an increase in the area for the high lifetime lipid domains. This implies that cholesterol increases the gel/L_o_ domains in OML. The same was also evident from a significant increase in the average fluorescence lifetime of Laurdan (Figure S3). In order to gain further insights into the changes in the Laurdan lifetime dynamics with cholesterol addition, we looked at the two distinct lifetime components.

For both, an increase in the average fluorescence lifetime was observed (Table 1). Interestingly, we observed a 2-fold increase in the faster (low) lifetime component (τ1) with cholesterol addition. This indicates that Chol increased the order and slowed the relaxation of Laurdan in the *L*_*d*_ phase by a larger magnitude compared to the slower (high) lifetime component (reflective of ordered/*L*_*o*_/gel domains).

Previous studies have reported that the lower lifetime values (2–3 ns) correspond to *L*_*d*_ like domains while higher lifetime values (> 4 ns) correspond to *L*_*o*_–*L*_*d*_ phase coexistence or gel phase domains.^[21,22]^ This suggests that exogenous Chol partitions preferentially into the *L*_*d*_ phases of OML. On the other hand, it is also conceivable that addition of exogenous Chol induces membrane lateral re-organization accounting for the above observations rather than partitioning behavior. At present, we cannot ascertain which factor is the major contributing factor. Overall, this experiment suggests that Chol modifies the phase state behavior, increases the overall order (deceases hydration) of OML by plausibly higher partitioning in *L*_*d*_-like fluid phases. Preferential interactions of exogenous Chol with OML lipids constituting the *L*_*d*_-like phase could account for this observation, but needs further investigation.

Next, AFM was used to orthogonally explore the topographical and lateral organization of solid supported bilayers (SSB) of OML along with *z*-height differences (Δ*h*) between *L*_*o*_–*L*_*d*_ phases.^[23,24]^ Consistent with our FLIM results, we observed phase separation in all the 3 lipid systems (Figure 3C). In case of OML only, Δ*h* between the two phases (*L*_*o*_–*L*_*d*_) was ~8 nm (Figure 3D) in accord with previous reports.^[20]^ This is attributed to the presence of glycolipids such as GPLs (Glycopeptidolipids), TDM (Trehalose dimycolate), and, MA (Mycolic acid) etc., with very long acyl chain lengths (C_60_–C_90_).^[25,26]^ SSB of OML+ 5 mol % Chol showed a slight reduction in Δ*h*, and, with 15 mol% Chol, it further reduced to ~6 nm. The Δ*h* reduction is likely driven by the incorporation of Chol molecules in both *L*_*d*_ and *L*_*o*_ region, thereby reducing the height mismatch between the domains, which otherwise is energetically unfavorable. This is in agreement with previous reports, where Chol has been shown to modulate the phase state behavior of phospholipids by decreasing the gel-*L*_*d*_ or *L*_*o*_–*L*_*d*_ phase height differences.^[24,27]^ Next, nanomechanical property of these SSBs were investigate via means of measuring breakthrough force (BrF), i. e., the force experienced by the cantilever tip to pierce through the bilayer.^[28]^ It is an indicator of the overall stiffness of the lipid bilayer. The BrF was calculated using approximately 200 force curves per sample/condition. The BrF for OML bilayer was around 1.80 nN (Figure S4), in agreement with our previous study.^[20]^ With the addition of 15 mol% exogenous Chol, two different distributions were observed which were fitted with Gaussian functions; a lower value centered around 3.75 nN and a higher value around 6.14 nN was observed. The increase in BrF indicates that cholesterol stiffens the OML bilayer by possibly getting incorporated in both *L*_*o*_ and *L*_*d*_ phases of OML nascent bilayer. This experiment also suggests that exogenous Chol alters the phase state behaviour of OML by reducing the *L*_*o*_–*L*_*d*_ height difference and increasing the stiffness of the OM. At present, we have no molecular details about the identity of individual *Msm* OML lipids partitioning to *L*_*o*_ *vs L*_*d*_*-*like phases and how that partitioning is impacted by exogenous Chol. It would require more specific experimentation (such as partitioning coefficients of purified lipids in model membranes that are known to form *L*_*o*_ *vs L*_*d*_ phases) coupled with molecular simulations to shed light on the same.

### Understanding the cholesterol partitioning into OML

**Figure 4:**
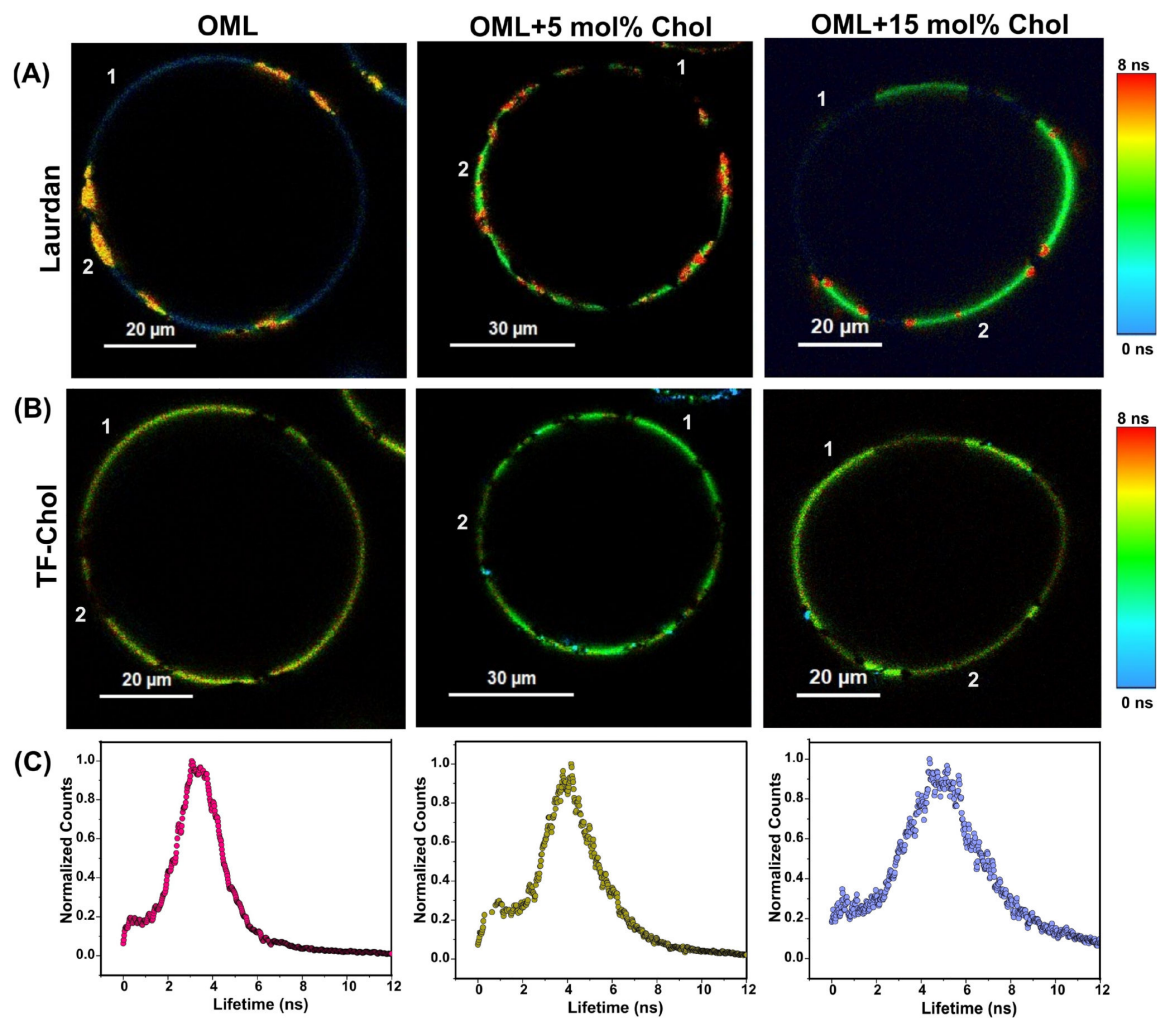
Understanding the cholesterol partitioning into the OML. Representative FLIM images for the indicated lipid system using two fluorescent probes, i. e., Laurdan (panel A) and Top-Fluor Chol (panel B). Panel C corresponds to the fluorescence lifetime distribution histogram of GUVs labelled with Top-Fluor Chol (TF-Chol). TF-Chol was excited using 440 nm diode laser and emission was collected using 532 long pass filter. Using a single vesicle, two different images were recorded for two different probes. Region 1 corresponds to *L*_*d*_ like domains, Region 2 corresponds to *L*_*o*_ like domain. All the measurements were carried out at 23 ± 1 °C.

To further ascertain in which phase of OML (*L*_*o*_ or *L*_*d*_) the exogenous Chol actually prefers to partition, we performed FLIM with both Laurdan and Top-Fluor Chol (TF-Chol), Figure 4. Top-Fluor Chol is known to partition preferentially only into the *L*_*o*_ phase or cholesterol-rich lipid phases,^[20,23]^ while Laurdan partitions uniformly in both phases of segregated lipid membranes.^[29]^ TF-Chol is a fluorescent cholesterol analogue, structurally distinct from cholesterol, but has been shown to be one of the best cholesterol-based probe displaying a near similar partitioning behavior and ordering properties as unlabeled Chol (ref [4] of Rev comments).^[30,31]^

Coexistence of *L*_*o*_, *L*_*d*_ and gel phases in OML was seen with lifetime segregated lipid domains using Laurdan FLIM (Figure 4A). However, the same vesicles demonstrated a near uniform distribution of Top-Fluor Chol barring a few regions (Figure 4B). These regions (region 2, Figure 4B) could possibly be the gel phase domains wherein we have earlier shown that Top-Fluor Chol cannot partition.^[20]^ Also, as expected, these Top-Fluor Chol-devoid gel phase domains exhibited the highest lifetime of Laurdan (Figure 4A) arguing for slowest Laurdan relaxation and hence underscoring presence of gel like lipid domains.

Next, in OML GUVs with exogenous Chol, we observed higher pixel counts of Top-Fluor Chol in regions that exhibited low Laurdan lifetime (region 1, Fig 4), i. e., fluid/disordered or *L*_*d*_ like regions. This suggest that exogenous Chol partitions into these fluid regions accessed by monitoring TF-Chol. This is mainly attributed to the known behavior of Chol to partition into both *L*_*o*_ and *L*_*d*_ phases in model and natural membranes.^[22,32]^ Such partitioning behavior would increase the order of the *L*_*d*_-like phases (aligning with Figure 2 A–B) and enable Top-Fluor Chol to partition favorably. Interestingly, we also observed significant but lower counts of Top-Fluor Chol pixels from regions corresponding to higher Laurdan lifetime (region 2, Fig 3B). This means that Chol partitions into the *L*_*o*_ or gel phase domains of OML as well, albeit lower than in *L*_*d*_, and makes these domains accessible for TF-Chol insertion, which otherwise were inaccessible (Figure 4A).

Then, we also selectively calculated the TF-Chol lifetime for regions 1 & 2 (Table 2); which corresponds to fluid and ordered lipid domains respectively and observed a steady increase in the lifetime with Chol addition. Overall, given the known *L*_*o*_-partitioning pattern of Top-Fluor Chol and the above results including AFM, it can be stated that exogenous Chol partitions into both *L*_*o*_ and *L*_*d*_ phase of OML, with likely higher preference for fluid regions. Top-Fluor Chol is also used as a probe to measure membrane viscosity.^[33]^ In line with this, an increase in the average lifetime of Top-Fluor Chol with the addition of cholesterol (Fig S5) indicated that the overall viscosity of OML increases.

**Figure 5:**
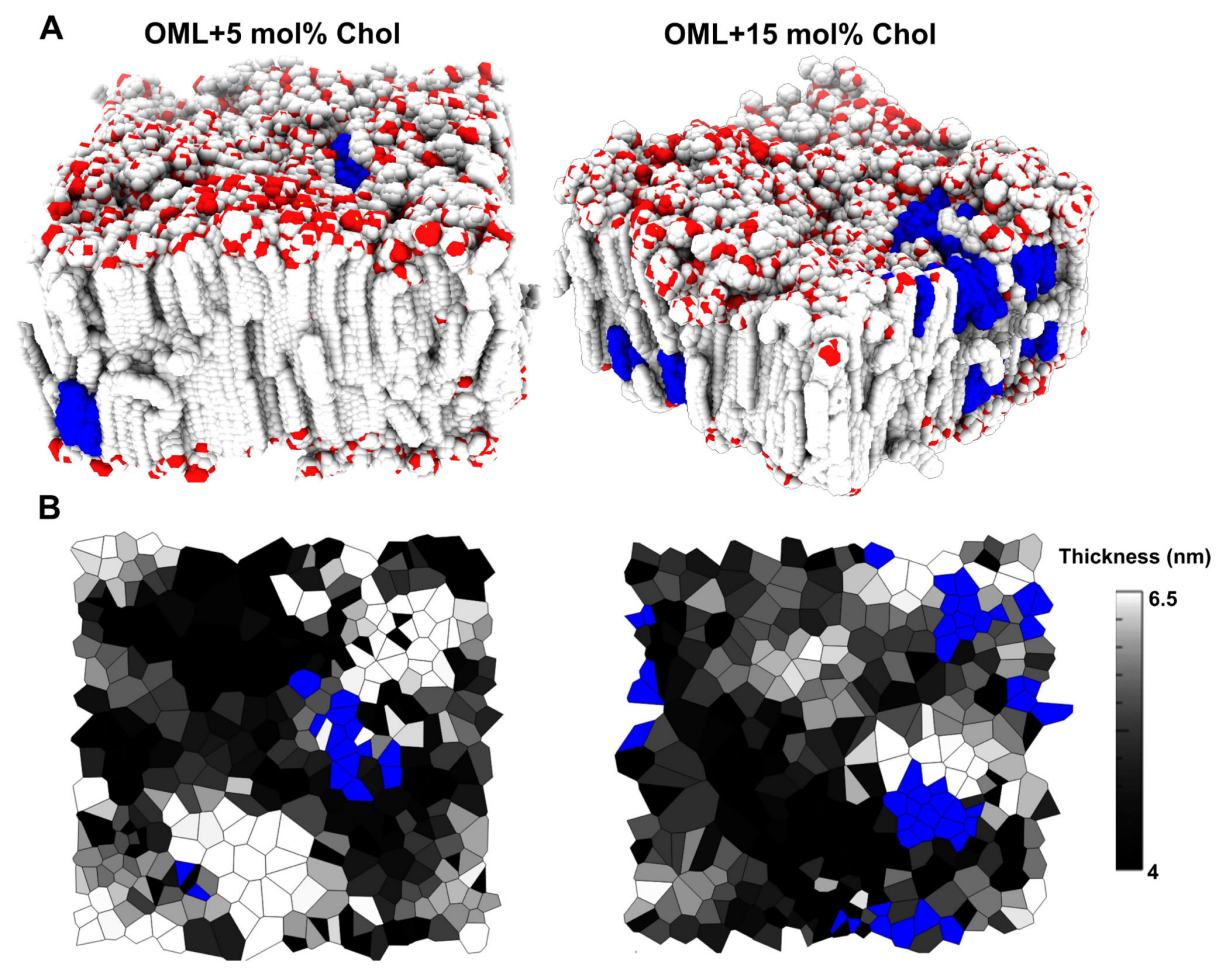
Understanding the cholesterol partitioning into the OML (A) Snapshots of molecular model of OML with addition of 5 and 15 mol% cholesterol. Gray colour-OML, Blue Colour-Cholesterol, red colour represents oxygen atoms in OML lipids. (B) Membrane thickness of OML at different cholesterol concentrations displaying height heterogeneity and preferential location of exogenous Chol in the thinner membrane regions and at the phase boundary of thinner and thicker membrane regions.

**Figure 6:**
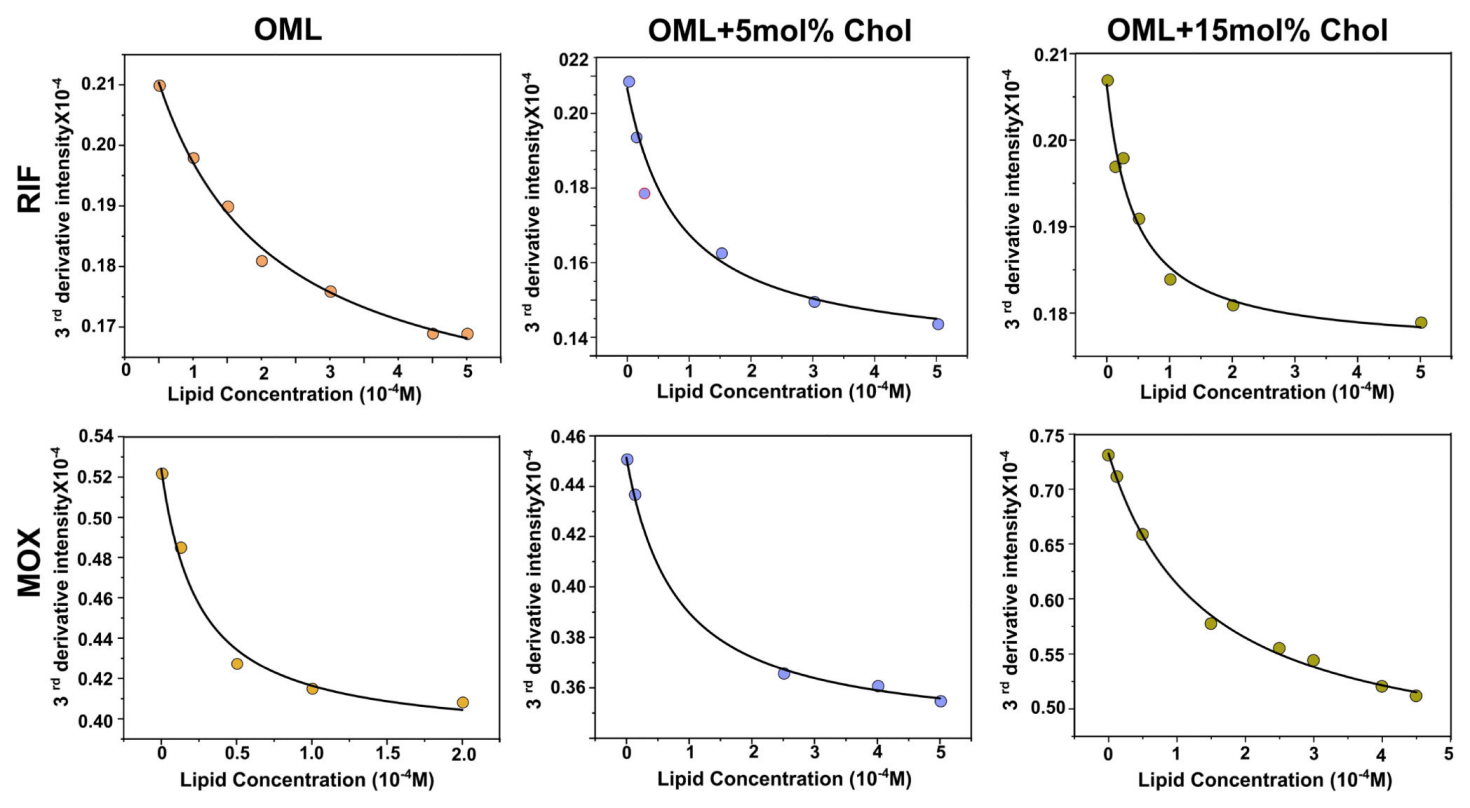
Representative Nonlinear least square regression curves of the 50 μM Rifampicin (RIF) and 30 μM Moxifloxacin (MOX) in OML obtained using third derivative UV absorption intensities measured using UV-visible spectroscopy. All the measurements were carried out at 37 ± 1 °C.

We also generated independent insights on the preferential location sites of exogenous Chol using previously defined molecular models of *Msm* OML (refs).^[34]^ Chol either clustered together and localized within the disordered region (the thinner region of the membranes) in line with AFM and FLIM results, but also at the interface of *L*_*o*_-*L*_*d*_; ordered and disordered region. This could reduce the line tension at the phase boundary and stabilize the phase-segregated OML bilayer (Figure 5A). The membrane thickness maps of OML with cholesterol also point to the same preferential localization sites of Chol (Figure 5B). Further, analysis of the area per lipid of OML with and without Chol (Figure 6) revealed an ordering effect of Chol on the OML membrane (supporting Fig 2), however had no significant effect on the diffusion of OML (Fig S7). This also aligns with a marginal increase or decrease in the order or fluidity of OML in the presence of exogenous Chol.

### Impact of exogenous cholesterol on drug partitioning in Msm OML

At particular points in the infection process, it has been observed that the ingested mycobacteria imports Chol from the host either via its *mce* transporters or by passive diffusion through cell envelope.^[10,11]^ This recruitment is most prominent during the latent stage of infection, wherein the intracellular bacteria need to shift bases to alternate sources of carbon for persistence. There is a tempting hypothesis that this exogenous Chol in the *Mtb* membranes during this phase may aid the bacterium in evading drug action and contribute to drug resistance and failed or limited treatment options for latent TB.^[18,19]^ Thus, we sought to correlate the impact of exogenous cholesterol on *Msm* OML-drug interactions.

Pharmacological activity of drug is often strongly correlated with its lipophilicity and it is normally represented as the logarithm of the n-octanol: water partition coefficient.^[35,36]^ Using spectral characteristics (λ_max_) of drug molecule, which changes when it transfers from an aqueous to lipid medium, one can quantify drug partitioning by calculating log D or *K*_*p*_ values.^[37]^ A higher value of log D indicates higher penetration of drug into the liposome^[38,39]^ and vice versa. We chose Moxifloxacin (MOX) that belongs to the fourth-generation fluoroquinolone antibiotics and is one of the components for treating MDR (Multidrug resistant)-TB.^[40]^ A decrease in log D for MOX in OML in presence of Chol indicated decreased partitioning of MOX into the OML (Table 3, Figure 5). This is expected given that exogenous Chol increases the overall order/packing of OML which mitigates the membrane partitioning of the rather hydrophilic MOX (with a logP value of 0.01).^[41]^ To confirm the same, we then tested Rifampicin, which is a known hydrophobic drug (with a logP value of 3.85)^[41]^ and saw an increase in the log D for Rifampicin (RIF) indicating an increase in partitioning in OML in presence of Chol (Table 3, Figure 5). This suggests that increased packing mediated by exogenous Chol in OML governs the drug-partitioning behavior dictated by the physiochemical characteristics of the drugs. To further validate the results, we performed an in-vitro viability assay of *Msm* using resazurin (Fig S8). We observed that the relative bacterial viability/cell growth was reduced in the presence of RIF when the bacteria were cultured and grown in cholesterol rich media. However, under similar growth conditions, MOX did not affect the viability. These data also support to the findings, which demonstrate attenuated activity of MOX in targeting persistent bacteria,^[18]^ likely driven by its attenuated partitioning in cholesterol-enriched bacterial cell membrane. Interestingly, it provides molecular insights demonstrating how RIF shows a unique bactericidal activity against persistent *Mtb* experimentally observed using in vitro and cell culture systems, and is attributed to its higher partitioning in bacterial membranes, especially in presence of host Chol.^[42]^

## Conclusions

The hallmark of *Mycobacterium tuberculosis* (*Mtb*) pathogenesis is its capability to survive under a range of antimicrobial environments. *Mtb* can survive for decades inside the host in the dormant state and it has the ability to reactivate, when the immune system of the host weakens. At present, there is no effective medication that eradicates dormant latent stage *Mtb* and hence understanding membrane dynamics of mycobacterium becomes extremely important to spur discovery of membrane-centric drugs. In the latent stage of infection, *Mtb* can hijack host lipids and utilize them as a nutrient source. One of these is cholesterol that enters into the bacterium via both *mce*-operated active transport as well as passive diffusion through cell envelope. This led us to investigate how cholesterol would alter the membrane dynamics of mycobacterium and subsequently remodel membrane-drug interaction landscape. Our work demonstrated that Chol modifies phase state behavior of outer membrane lipids of *Msm*. It gets incorporated into both *L*_*o*_ and *L*_*d*_ phase, with higher preference for the latter, and increases the overall order of the system both at the headgroup region and acyl chain region. The exogenous Chol rewires drug partitioning behavior of structurally dissimilar anti-TB drugs, suggesting a higher clinical potential of use of more hydrophobic drugs to target persistent, late infection state intracellular bacteria.

## Supplementary Material

Supplementary material

## Figures and Tables

**Table 1:** Fluorescence lifetime values of Laurdan obtained after lognormal fitting of lifetime distribution histogram for the above-mentioned lipid systems.

Sr No.	Lipid System	τ1 (ns)	τ2 (ns)
1	OML	2.41 ± 0.01	6.79 ± 0.01
2	OML + 5 mol% Chol	4.45 ± 0.02	7.23 ± 0.01
3	OML + 15 mol% Chol	5.38 ± 0.01	7.81 ± 0.02

**Table 2:** Using GUVs from fig 4, fluorescence lifetime values of TF-Chol for region 1 (*L*_*d*_ like domains) and region 2 (*L*_*o*_ like domains) were extracted for the below-mentioned lipid system.

Sr No.	Lipid System	Fluorescence lifetime of region 1 (ns)	Fluorescence lifetime of region 2 (ns)
1	OML	3.6 ± 0.34	2.9 ± 0.02
2	OML + 5 mol % Chol	4.8 ± 0.18	3.3 ± 0.04
3	OML + 15 mol % Chol	5.3 ± 0.27	3.7 ± 0.10

**Table 3:** Log D values of Rifampicin calculated from the fitted curves in figure 5 for the respective lipid systems. Data are representative of three-five independent experiments as mean ± SD. All the measurements were carried out at 37 ± 1 °C.

Sr No.	Lipid System	Log D for RIF	Log D for MOX
1	OML	3.71 ± 0.14	4.78 ± 0.33
2	OML + 5 mol% Cholesterol	4.13 ± 0.08	3.94 ± 0.13
3	OML + 15 mol% Cholesterol	4.26 ± 0.13	4.09 ± 0.09

## Data Availability

The data that support the findings of this study are available from the corresponding author upon reasonable request.
