# Supplementary material for: Effect of Host Cholesterol on the Membrane Dynamics of Outer Membrane Lipids of Mycobacteria
Source: Chem Asian J. Author manuscript; Available in PMC 2024 Dec 8. (PMC7616960; doi:10.1002/asia.202300697)
Supplement: Supplementary material [file EMS195555-supplement-Supplementary_material.pdf]

Chemistry - An Asian Journal

Supporting Information

# Effect of Host Cholesterol on the Membrane Dynamics of Outer Membrane Lipids of Mycobacteria

Pranav Adhyapak, Kuan Liang, Mojie Duan, and Shobhna Kapoor*

# Supporting Information

**This SI file contains methods and materials, 8 SI Figures and SI references.**

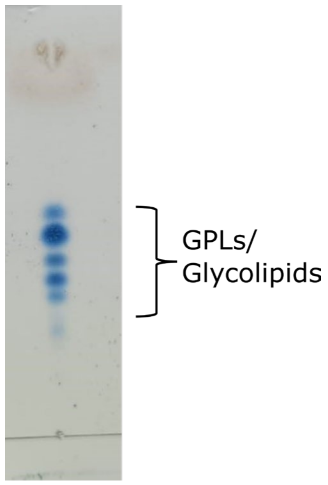


**Figure S1**: TLC profiles of outer membrane lipids extracted from *Msm* developed in chloroform-methanol (9.5:0.5). Glycolipids (Blue colored spots) were visualized using anthrone reagent.

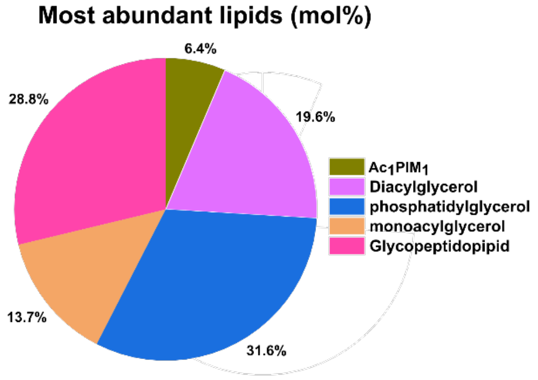


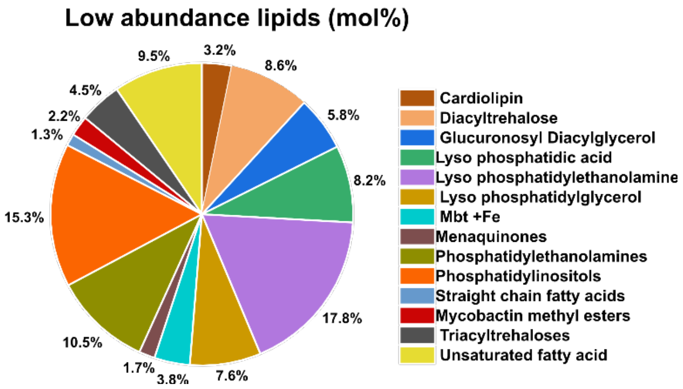


**Figure S2**: Qualitative and quantitative distribution of outer membrane lipids calculated using LC-MS/MS. Reserpine (1ppm) was used as an internal standard. Data is representative of mean of 3 experiments.

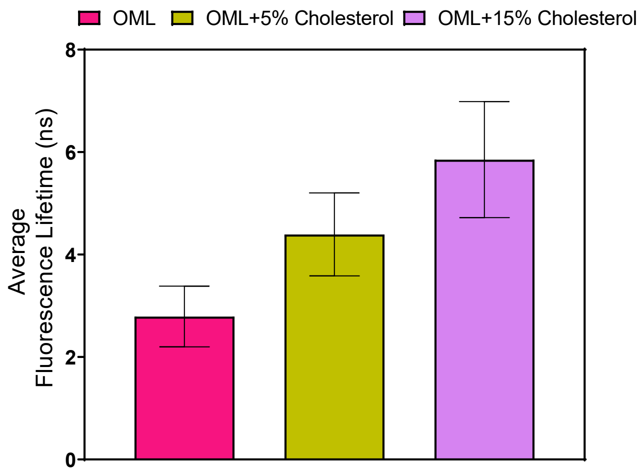


**Figure S3**: Average fluorescence lifetime of Laurdan obtained from FLIM experiment of OML GUVs with or without addition of 5 and 15 mol% cholesterol. Data is representative of mean ± SD of 30 vesicles (for each data set) of three independent experiments. All the measurements were carried out at 23 ± 1ºC.

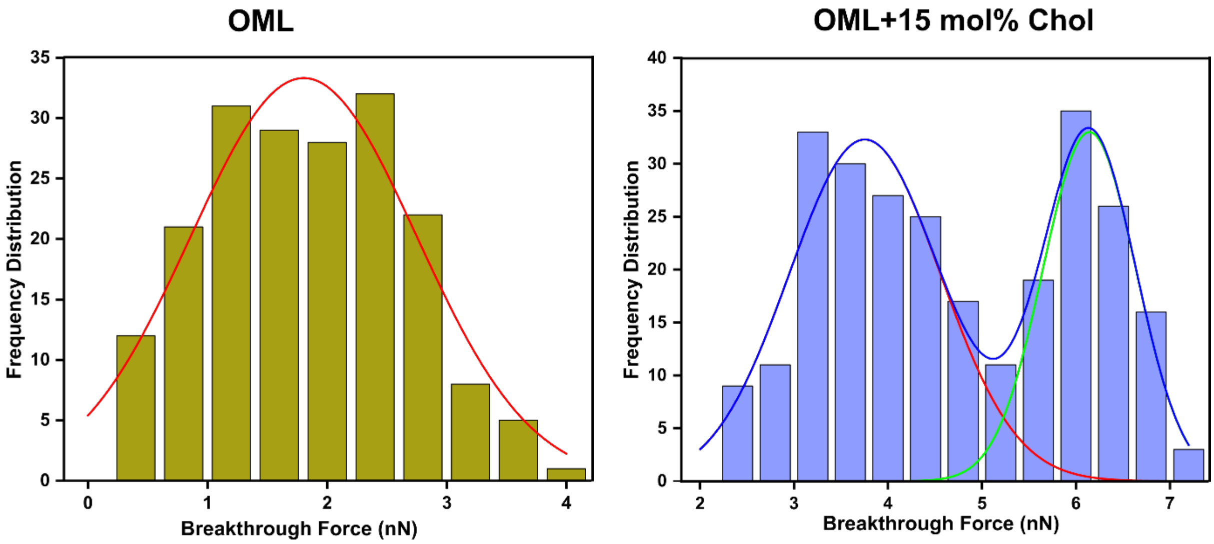


**Figure S4**: Breakthrough force distribution (BrF) of the indicated lipid systems recorded using AFM force spectroscopy of solid supported bilayers. The BrF distribution was fitted with Gaussian fitting function in Origin Pro 2018b. The BrF distribution shows the mean values centered at 1.80 ± 0.07 nN for OML. In case of OML+15 mol% Chol, 2 distinct BrF distributions were observed with one centered at 3.75 ± 0.08 nN and other at 6.14 ± 0.06 for OML+15 mol% Chol. Data is representative of mean ± SD of 196 and 279 force curves recorded for OML and OML+15 mol% Chol respectively. Data is obtained from 3 independent experiments. All the measurements were carried out at 23 ± 1 ºC.

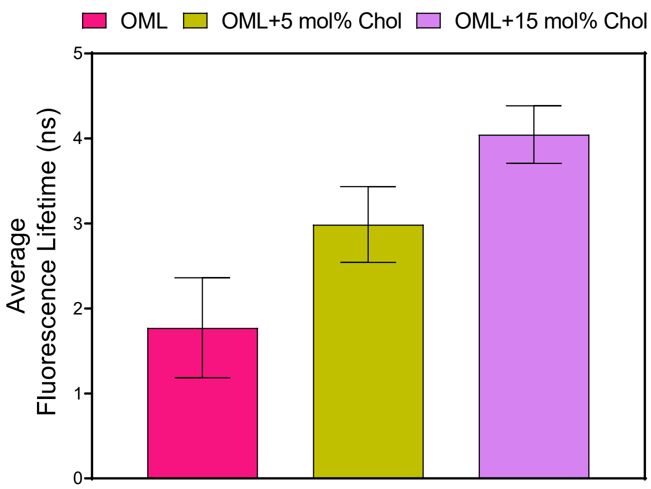


**Figure S5:** Average fluorescence lifetime of TF-Chol obtained from FLIM experiment of OM GUVs with or without addition of 5 and 15 mol% cholesterol. Data is representative of mean ± SD of 5 vesicles (for each data set) of two independent experiments. All the measurements were carried out at 23 ± 1 ºC.

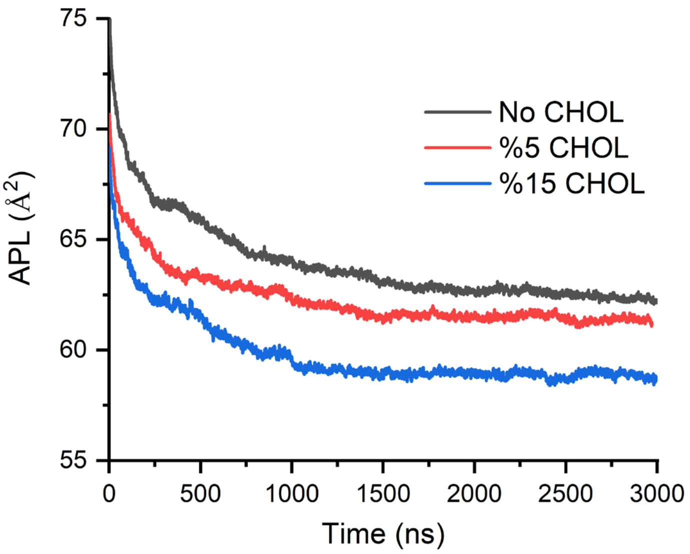


**Figure S6:** The average area per lipid in the above-mentioned membrane models as a function of simulation time.

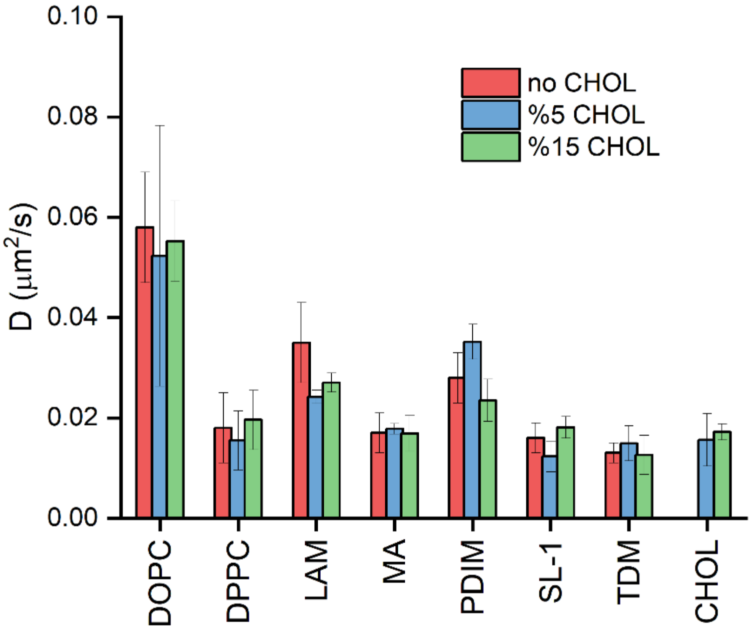


**Figure S7:** Diffusion coefficient of the representative lipids present in OML.

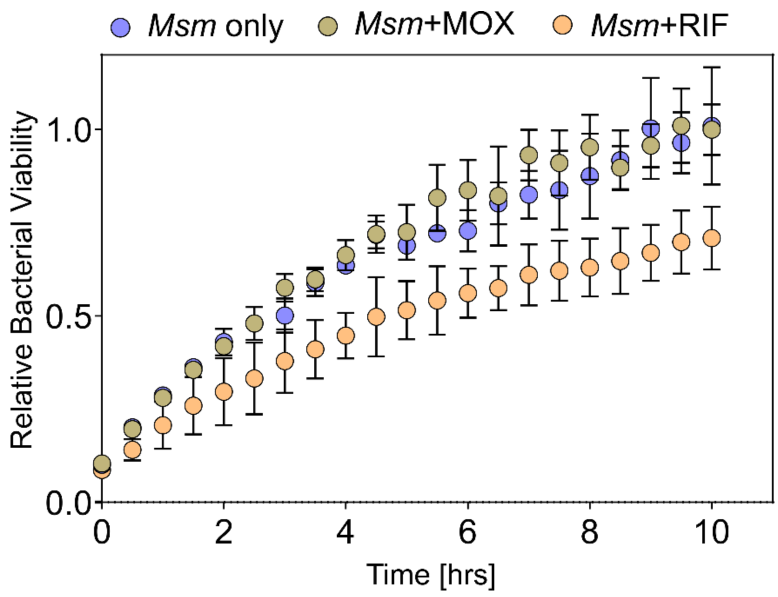


**Figure S8:** In vitro viability assay of *Msm* in the presence of MOX (0.2 μM) and RIF (3 μM). The bacteria were grown on 250 μM cholesterol. Data is representative of mean ± SD of 3 experiments.

## Materials & Methods

### Materials:

The fluorescent probes Laurdan (1-(6-(dimethylamino) naphthalen-2-yl) dodecan-1-one) and DPH (1,6-Diphenyl-1,3,5-hexatriene) were purchased from Sigma-Aldrich. Other chemicals such as $MgCl_2$ and Nacl were procured from Merck. Cholesterol was purchased from TCI chemicals. Tris and HEPES were obtained from MPI and Himedia respectively. Top Fluor Cholesterol (23-(dipyrrometheneboron difluoride)-24-norcholesterol) was purchased from Avanti Polar Lipids. Spectroscopic grade chloroform was purchased from Spectrochem. Rifampicin was obtained from Medchem express.

### Extraction of Lipids

Lipids were extracted selectively from outer membrane (OM) of *M.smegmatis* based on a previously reported method[1]. For the recovery of all non-covalently bound OM lipids, 10mg dry weight of cells was extracted with 1mL of RMS (10mM sulfosuccinic acid 1,4bis (2ethylhexyl) ester sodium salt (AOT) in heptane). The extraction was carried out in monophasic solutions. The outer membrane lipids were then purified from AOT using neutral alumina-based column chromatography[2]

### Preparation of Liposomes:

Liposome preparation of different combinations was carried out using a gentle hydration method. Briefly, 0.5 mg lipid was used in each experiment and a 1:200 (Laurdan: Lipid) ratio was maintained for the TCSPC experiment and a 1:100 (DPH: Lipid) ratio was maintained for the anisotropy experiment. The lipid solutions were then dried under a stream of nitrogen gas followed by lyophilization overnight. The dried films were hydrated using 1 ml of 20 mM Tris, and 5 mM $MgCl_2$ (pH-7.4) and sonicated in a water bath at 65$^{o}$C for 10 min, followed by 5 freeze-thaw cycles to generate large unilamellar vesicles (LUVs). For the drug (rifampicin) partitioning study, liposomes were passed through 100 nm Nucleopore® Polycarbonate Track-Etch™ Membranes (Whatman GmbH, Dassel, Germany) using a Mini-Extruder at 70 $^{o}$C to obtain small unilamellar vesicles. Also, for drug partitioning work, 10 mM HEPES, and 150 mM Nacl buffer (pH 7.2) were used.

**Steady State and Time-Resolved Fluorescence Measurements:**

The steady-state anisotropy measurements were performed on Horiba Scientific Fluoromax 4 fluorescence spectrophotometer with a temperature controller having the accuracy of ±0.1 ºC. DPH was excited at 352 nm and anisotropy was calculated at 430 nm emission peak. Anisotropy was calculated according to the following equation.

$$r = \frac{I_{\parallel} - GI_{\perp}}{I_{\parallel} + 2GI_{\perp}} \tag{1}$$

Where, $G = \frac{I_{\parallel}}{I_{\perp}}$ (2)

Fluorescence decay was recorded using a DeltaFlex time-correlated single-photon counting (TCSPC) system (HORIBA Scientific). Samples were excited at 360 nm with a Titanium Sapphire laser source. Emission decays were measured at 430 nm (~80 ps pulse width, 80 MHz repetition rate). For each emission wavelength, the acquisition time was 100 ns. A Ludox solution was used to record the instrument-response function. The emission was collected at a magic angle (54.7°) polarization. All measurements were performed at 23 ± 1ºC. Fluorescence decay traces were fitted to exponential or sum of exponential functions according to equation 1 using iterative deconvolution in EzTime software.

$$I_{\lambda}(t) = I_0(\lambda) \sum_i a_i\, e^{-t/\tau} \tag{3}$$

where $\tau_i$ and $a_i$ are the lifetime and amplitude of the $i^{th}$ component, respectively.

**Preparation of Giant Unilamellar Vesicles (GUVs):**

For GUV preparation, 0.5 mg lipid was used in each experiment. Laurdan and Top Fluor Cholesterol were added to the lipid sample to yield a final probe: lipid ratio of 1:100. GUVs were prepared using the electroporation method in a temperature controlled custom made chamber using optically transparent and electrically conductive indium tin oxide (ITO) coated glass coverslips (SPI supplies, USA). 100 µL of lipid mixture was spin-coated (1000 rpm for 30 sec) onto the coverslips and subsequently dried under vacuum overnight. The coverslips were placed within the electroporation cell and the cell was filled with Tris-$MgCl_2$ buffer. Lipids were hydrated within the cell at a temperature of 65°C and a low-frequency AC field (sinusoidal wave

function with a frequency of 10 Hz and an amplitude of 2Vp-p) was applied for 90 min. The cell was gradually cooled to room temperature.

**Fluorescence Lifetime Imaging and Microscopy (FLIM):**

FLIM was performed on a MicroTime 200 (Picoquant, Gmbh, Germany) using Time-tagged Time-resolved (TTTR) methodology. This setup is attached to an inverted microscope (IX71) equipped with a water immersion objective (UPlan SApo NA 1.2, 60X, WD = 0.28 mm). Laurdan and Top Fluor Cholesterol in GUVs were excited at 405 nm and 440 nm respectively using a pulsed diode laser. Laurdan fluorescence emission was collected using a 460/60 bandpass filter and Top Fluor Cholesterol fluorescence emission was collected using a 532 nm long pass filter (AHF/Chroma, Germany). The collected fluorescence signal was directed to a single photon avalanche photodiode (SPAD) detector. The images were acquired with 512X512 pixel resolution. The fluorescence decays were analyzed using the inbuilt software of MicroTime 200 and were fitted using iterative deconvolution, to bi-exponential function.

$$I_\lambda\ (t) = I_0\ (\lambda)\ \sum_{i-1}^{n} a_i\, e^{-t/\tau_i} \qquad (4)$$

where I(t) is the intensity of the fluorescence at time t, $a_i$ is the pre-exponential factor for the fraction of the fluorescence intensity, $\tau_i$ is the fluorescence lifetime of the emitting species, and n is the number of exponentials used. The average fluorescence lifetime was calculated using the following relation.

$$\tau_{avg} = \frac{\alpha_1\tau_1 + \alpha_2\tau_2}{\alpha_1 + \alpha_2} \qquad (5)$$

where $\alpha_1$ and $\alpha_2$ are the pre-exponential factors representing the fractional contribution of the decaying component with a lifetime $\tau_i$.

Deconvolution of the fluorescence lifetime distribution curve was performed using Origin Pro 2018b. All the fitting equations were utilized with lognormal function as previously described[3,4]

$$y = y0 \frac{A}{\sqrt{2\pi} wx} e^{\frac{-[ln\frac{x}{xc}]^2}{2w^2}} \quad (6)$$

where *y0* is offset, *w* is log-standard deviation, *A* is an area, and *xc* is the center. Using fitting acquisition, the integrated area and its area ratio (%) were obtained. Two or Three-peak fitting was performed for the respective curves and the adjusted $R^2$ value, demonstrating the goodness of fit, ranged from 0.994 to 0.998.

**Atomic Force Microscopy:**

AFM Imaging was carried out in Intermittent contact (AC) mode and force spectroscopy was done in contact mode with MFP-3D atomic force microscope (Asylum Research, Santa Barbara, CA, USA.) For Imaging and force, spectroscopy of model membranes, silicon nitride cantilevers with spring constants 0.03-0.26 N/m were used.

For the preparation of supported lipid bilayer (SLBs), liposomes were prepared as mentioned earlier and then passed through 100 nm Nucleopore® Polycarbonate Track-Etch™ Membranes (Whatman GmbH, Dassel, Germany) using a Mini-Extruder at 70 °C to obtain small unilamellar vesicles. (Avanti Polar Lipids Inc, Alabaster, AL, USA). Ted Pella mica (9.9 mm in diameter) was glued on a 60 mm petri-dish and the mica was cleaved with scotch tape to form an atomically flat surface. Then, 2 ml of Tris-$MgCl_2$ buffer and 1 ml of liposome suspension were added to the mica and incubated for 2 hours in a water bath at 65 °C. After incubation, the sample was washed thoroughly to remove any unfused liposomes. The typical scan rate for images was 0.5 Hz. Images were recorded at various locations on the mica. Up to five images were recorded at various locations on SLB. The images were plane fitted at order zero and flattened at order 1. Force spectroscopy was performed to calculate the resistance experienced by the cantilever to rupture the bilayer. In this approach, force-distance curves were recorded. The supported lipid bilayer is considered a planar arrangement of adjacent lipid molecules. As the tip approach and indent the bilayer, the latter gets compressed, the molecular area underneath the tip changes, and lipid positions fluctuate. Between the adjacent molecules of lipids, energy is elastically stored until the rupture point occurs. This creates a jump in a force-distance curve, as the tip

pierces through the bilayer. This jump is considered a breakthrough force (BrF) event5. Cantilever velocity was maintained at 0.7-1μm/s and the applied force was 15 nN. Force maps were recorded in 16X16 pixels.

**Determination of Partition Coefficient (*$K_p$*):**

The partition coefficient ($K_p$) of rifampicin between the aqueous buffer and lipid vesicle was determined using the UV-visible spectrometry technique6,7. Briefly, 50 μM rifampicin/30 μM moxifloxacin was added to increasing concentration (0-500 μM) of lipid vesicles and incubated at 37°C for one hour and the absorption spectra were recorded using a multi detection microplate reader (ThermoFisher) from 200-700 nm with 2 nm interval at 37 °C. For the calculation of *Kp* values, third derivative intensities were considered by fitting the experimental data to the following equation by nonlinear regression method using Origin 2021a.

$$D_T = Dw + \frac{(Dm - Dw)K_P[L]Vm}{1 + K_p[L]Vm} \tag{7}$$

Where, D represents the second derivative intensity$\left(D = \frac{d^3abs}{d\lambda^3}\right)$ obtained from the absorbance of the total concentration of drug ($D_T$), rifampicin distributed in the aqueous phase ($D_w$), rifampicin distributed in lipid membrane phase ($D_m$), [L] is the molar lipid concentration and $V_m$ is the molar volume reported previously[7].

**Mass Spectrometry Analysis:**

Lipids were separated using a 1290 Infinity Ultra-High-Performance Liquid Chromatography System and an 6545XT AdvancedBio Quadrupole Time-of-Flight system (Agilent Technologies, Santa Clara, CA) was used for the MS/MS analysis of the extracted lipids. The lipids were identified using databases available online.[8,9] Three independent replicates were performed for all measurements.

**Bacterial Cell Viability Assay:**

*Msm* cells were grown on M7H9 medium and instead of glycerol, 250 μM cholesterol was used as a carbon source. $OD_{600}$ of *Msm* was adjusted to 0.1 and cells were incubated with 0.2 μM MOX or 3 μM RIF with 10 μl of 0.02% resazurin for 24 hours.

All the experiments were performed in duplicates in a 96 well plate at 37°C. The absorbance of resazurin and resorufin was recorded at 600 nm and 570 nm respectively in a CLARIOstar^plus (BMG LABTECH) microplate reader and the relative cell viability was calculated as reported previously.[10]

**MD Simulations:**

The lipid force fields parameters and MD simulation setting are similar to our previous work.[7,11] For cholesterol and lipids DOPC and DPPC, the force field parameters are achieved from Lipid14.[12] For the other lipids, the partial charge by fitting the electrostatic potentials through the restrained electrostatic potential (RESP) method[13] by the antechamber module.[14] The atom types, bonded interaction parameters and Van der Waals interaction parameters were defined by the lipids Generalized Amber Force Field (GAFF).[15] The lipid bilayer membranes were packed by the Packmol software.[16] The ratio of lipid components in the NO-CHOL outer/mycomembrane was SL-1:TDM:PDIM:LAM:DOPC:DPPC:MA = 10%:10%:10%:10%:15%:15%:30%. For the %5 and 15% cholesterol-OM systems, 30 cholesterol and 90 cholesterols were added into the membranes. The lipid number in all of the three systems were composed of 600 different lipid molecules. About 30,000 TIP3P water molecules were added to build the solvent box, sodium ions were added to neutralize the system. The steepest descent method was performed to minimize the system until the root-mean-square of energy gradient was < 0.0001 kcal/mol Å or the maximum iteration steps reached 10,000. The system was then heated to 300 K linearly in the periods of 100 ps in the NVT ensemble with the weak harmonic potential (10 kcal/mol Å) on the heavy atoms. Subsequently, a 1-ns unrestrained equilibration with Langevin thermostat[17] in the NPT ensemble were performed. The bonds involving hydrogen were constrained with the SHAKE algorithm. 3000-ns production runs were then carried out by CUDA-version Amber16[18] to equilibrate the membrane systems.

**SI References**

[1] R. Bansal-Mutalik, H. Nikaido, *Proc Natl Acad Sci U S A* **2014**, *111*, 4958–4963.

[2] P. Adhyapak, A. T. Srivatsav, M. Mishra, A. Singh, R. Narayan, S. Kapoor, *Biophys J* **2020**, *118*, 1279–1291.

[3] T. Parasassi, G. De Stasio, G. Ravagnan, R. M. Rusch, E. Gratton, *Biophys J* **1991**, *60*, 179–189.

[4] P. F. F. Almeida, W. L. C. Vaz, T. E. Thompson, *Biophys J* **1993**, 64, 399-412.

[5] S. J. Attwood, Y. Choi, Z. Leonenko, *Int J Mol Sci* **2013**, *14*, 3514–3539.

[6] L. M. Magalhães, C. Nunes, M. Lúcio, M. A. Segundo, S. Reis, J. L. F. C. Lima, *Nat Protoc* **2010**, *5*, 1823–1830.

[7] A. P. Menon, W. Dong, T. H. Lee, M. I. Aguilar, M. Duan, S. Kapoor, *ACS Bio and Med Chem Au* **2022**, *2*, 395–408.

[8] E. Layre, L. Sweet, S. Hong, C. A. Madigan, D. Desjardins, D. C. Young, T. Y. Cheng, J. W. Annand, K. Kim, I. C. Shamputa, M. J. McConnell, C. A. Debono, S. M. Behar, A. J. Minnaard, M. Murray, C. E. Barry, I. Matsunaga, D. B. Moody, *Chem Biol* **2011**, *18*, 1537–1549.

[9] M. J. Sartain, D. L. Dick, C. D. Rithner, D. C. Crick, J. T. Belisle, *J Lipid Res* **2011**, *52*, 861–872.

[10] L. Mathew, D. K. Verma, K. Liang, M. Duan, R. Dadhich, S. Kapoor, *ACS Appl Bio Mater* **2023**, 6, 3066-3073.

[11] P. Adhyapak, W. Dong, S. Dasgupta, A. Dutta, M. Duan, S. Kapoor, *Chem Asian J* **2022**, *17*, e202200146.

[12] C. J. Dickson, B. D. Madej, Å. A. Skjevik, R. M. Betz, K. Teigen, I. R. Gould, R. C. Walker, *J Chem Theory Comput* **2014**, *10*, 865–879.

[13] C. I. Bayly, P. Cieplak, W. D. Cornell, P. A. Kollman **1993**, 97, 10269-10280.

[14] J. Wang, W. Wang, P. A. Kollman, D. A. Case, *J Mol Graph Model* **2006**, *25*, 247–260.

[15] J. Wang, R. M. Wolf, J. W. Caldwell, P. A. Kollman, D. A. Case, *J Comput Chem* **2004**, *25*, 1157–1174.

[16] L. Martinez, R. Andrade, E. G. Birgin, J. M. Martínez, *J Comput Chem* **2009**, *30*, 2157–2164.

[17] S. Pal, S. Balasubramanian, B. Bagchi, *Phys Rev E* **2003**, *67*, 061502.

[18] D. A. Case, T. E. Cheatham, T. Darden, H. Gohlke, R. Luo, K. M. Merz, A. Onufriev, C. Simmerling, B. Wang, R. J. Woods, *J Comput Chem* **2005**, *26*, 1668–1688.
